# Long-Term Saline Water Adaptation Alters the Meat Quality of *Micropterus salmoides* from a New Salt-Tolerant Population

**DOI:** 10.3390/foods14183180

**Published:** 2025-09-12

**Authors:** Caixia Lei, Hanru Song, Peng Wang, Hongmei Song, Jingxin Du, Tao Zhu, Jing Tian, Shengjie Li

**Affiliations:** 1 Key Laboratory of Tropical and Subtropical Fishery Resources Application and Cultivation, Ministry of Agriculture and Rural Affairs, Pearl River Fisheries Research Institute, Chinese Academy of Fishery Sciences, Guangzhou 510380, China; 2 College of Life Science, Huzhou University, Huzhou 313000, China; 3 College of Marine Science, South China Agricultural University, Guangzhou 510642, China

**Keywords:** nutritional quality, sensory value, flesh texture, flavor compounds, salt-tolerance, *Micropterus salmoides*

## Abstract

Meat quality critically influences product value and consumer preferences. Here, the effect of long-term saline adaptation on flesh nutritional quality, sensory value, texture, and flavor was evaluated in a new *Micropterus salmoides* salt-tolerant population. The results showed that the salt-tolerant population exhibited decreased lipid, saturated fatty acid, and long-chain polyunsaturated fatty acid contents but increased monounsaturated fatty acid content, accompanied by upregulated elongase 5 and fatty acid desaturase 6 mRNA levels. The meat color of the new population was brighter and fresher, with a slightly less red tint, and the increased 2,3-butanedione content resulted in a desirable creamy aroma. 3-Pentanone was the most abundant volatile substance in both populations. Regarding taste parameters, the salt-tolerant population had improved hardness, stickiness, chewiness, resilience, cooking loss, myocyte space, and collagen content. Alanine, proline, and histidine were the main amino acids responsible for flavor presentation. The salt-tolerant population was found to have lower bitter and higher sweet amino acid contents. Higher umami nucleotides and lower pH intensified umami and sourness in salt-tolerant meat. The saltiness of the new-population meat was enhanced. This study comprehensively evaluated the flesh quality of a salt-tolerant *M. salmoides* population with the potential for cultivation, thereby providing a reference for its potential development as an alternative aquaculture strain.

## 1. Introduction

Fish serve as an important food source for humans, contributing > 20% of animal protein. In recent years, the number of people facing acute food crises has increased annually, reaching 232 million in 2023 [1]. It is predicted that the supply of aquatic animals must increase by 22% to meet the needs of the growing population by 2050, based on data from the World Fisheries Statistical Yearbook [2]. Saline–alkali land is a potential food-producing area that has not been fully explored, totaling 1125 million hectares worldwide [3], including approximately 99 million hectares in China [4]. The development of saline–alkali land has received considerable attention recently for mitigating food crises and developing economies, and saline–alkali water (water environments with high salinity and high alkalinity) aquaculture is an important topic in this field. The high salinity and alkalinity of saline–alkali water pose considerable challenges for fish, as this water negatively affects their growth, survival, and multiple physiological activities [5,6]. Naturally, a few native fish species (e.g., *Gymnocypris przewalskii* and *Leuciscus waleckii*) have evolved the ability to grow normally in saline–alkali water [3]. Some fish, including *Oreochromis niloticus* [7] and *Carassius auratus gibelio* [8], have been successfully cultivated in saline–alkali water. Recently, a new salt-tolerant population of *Micropterus salmoides* was also bred by our research team; the growth of this species in 9‰ salt water was 21.5% higher than that in an unselected group.

In addition to growth, the overall commercial value of a product is the primary consideration for producers. In recent years, consumers have stressed their desire for high-quality fish. Flesh quality is comprehensively determined by genetics, the water environment, feed composition, and feeding procedure [9]; it is a combined reflection of nutrient levels (e.g., protein, fatty acid, and vitamin levels), sensory qualities (e.g., color, pH, and smell), and taste (e.g., hardness and elasticity) [10,11,12]. Substantial information has been accumulated regarding fish flesh quality improvement, including dietary composition [13], stocking density [14], and culture models [15]. Studies on *Lateolabrax japonicus* [16] and Nile tilapia [9] have shown that salinity, which is an important physical and chemical characteristic of aquaculture water environments, affects flesh quality.

Previous studies have reported the benefits of appropriate salinity on muscle quality. In *Ctenopharyngodon idellus*, a slight increase in salinity improved meat quality, whereas negative effects were observed on the meat quality of individuals grown under high levels of water salinity [17,18]. Analogously, *Snakehead* grown under low levels of salinity showed higher muscle quality than those grown under high water salinity [19]. However, *Scylla paramamosain* cultured at low salinity exhibited a decrease in meat quality [20]. In *M. salmoides*, the optimum salinity for growth is reportedly 5.21‰ [21]. However, our new population was bred at 9‰ salinity, raising uncertainty regarding the effects of salinity on muscle quality, as the fish may adapt to the altered osmotic environment by adjusting its tissue biochemical structure. Moreover, the duration of salinity exposure is important. In the short term, decreased body moisture, reduced swimming frequency, and increased energy consumption are physiological responses of fish to salinity stress, as well as internal forces affecting muscle quality [22]. In contrast, a limited number of studies have focused on the effects of long-term adaptation to saline environments on flesh quality, to the best of our knowledge.

Therefore, this study aimed to determine the effect of long-term saline adaptation on *M. salmoides* flesh quality using a new salt-tolerant population. The results will enrich our understanding of the effect of a saline environment on fish flesh nutritional quality, sensory value, texture, and flavor, thus facilitating the development of saline–alkali water culture.

## 2. Materials and Methods

### 2.1. Fish and Sampling

The fish used for the experiments (of unknown sex) were provided by our partner, Liang Aquatic Seed Industry Co., Ltd. (Foshan, China); the weights of these fish are shown in Supplementary Table S1. The fish were sampled from a new population (the fourth generation) adapted to 9‰ salinity, as well as from a freshwater population (control group) that is currently widely farmed. The foundational parental stock for the salt-tolerant selection originated from the “Youlu 3” strain, which was selected by our team in 2018, with the control population being maintained under identical conditions and being sampled from the same genetic base population. The two populations were fed the same commercial feed (Haida Feed Group Co., Ltd., Guangdong, China) and were raised in the same facility. Individuals of the new salt-tolerant population were pre-implanted with electronic tags. Before sampling, the fish were fasted for 24 h and anesthetized with 0.01% (*w*/*v*) 2-phenoxyethanol (Sigma-Aldrich LLC., Shanghai, China). After approximately 5 min, the sampling was performed when the fish showed no response to external touch or stimuli and total loss of equilibrium. First, the middle areas of the dorsal and abdominal skin were selected for color detection after the moisture on the surface of the fish was dried. The muscles in the same areas were also used to detect pH. In addition, a 1 cm^3^-sized muscle sample centered around the lateral line was excised for texture analysis. Additionally, approximately 5 g (Supplementary Table S1) of fresh muscle samples was used for cooking loss determination and electronic tongue analysis, respectively. The remaining samples were stored at −80 °C or fixed in 4% paraformaldehyde (Solarbio Science & Technology Co., Ltd., Beijing, China).

### 2.2. Biochemical Analysis

Muscular crude protein, crude fat, ash, and moisture were measured using the methods of the Association of Official Analytical Chemists Society [23]. Crude protein content was calculated as N × 6.25 after digestion (98% sulfuric acid) using a Kjeldahl nitrogen apparatus (DRK-K616, Drick, Jinan, China). Crude fat was extracted with petroleum ether using a commercial tester (DRK-SOX316; Drick, Jinan, China). Moisture was measured using the drying method at 105 °C. The sample was subjected to 800 °C for 1 h and 550 °C for 8 h, after which the ash content was determined using the gravimetric method.

### 2.3. Fatty Acid Profile Analysis

The fatty acid composition of the flesh was measured as described in our previous study, with minor modifications [24]. Briefly, approximately 300 mg of the sample was used to extract total lipids using chloroform/methanol (2:1 by vol). Subsequently, distilled water was added, the mixture was centrifuged at 2500× *g* for 5 min after mixing, and the lower liquid was retained. The residual mixture was then extracted using chloroform. The obtained solution was dried at 45 °C in a water bath using a nitrogen blower (UGC-24C, Yousheng United Technology Co., Ltd., Beijing, China). The sample was then saponified by heating at 65 °C in a water bath for 1 h in potassium hydroxide methanol solution (0.5 mol/L) under gentle shaking conditions. After cooling, 15% (*v*/*v*) boron trifluoride-methanol solution was added, and the mixture was subsequently incubated at 75 °C for 30 min. Finally, n-hexane and saturated sodium chloride were added, and the upper layer was separated by centrifugation at 2500× *g* for 5 min. The fatty acid composition of the samples was investigated using GC (Agilent 7890B; Agilent Technologies, Santa Clara, CA, USA), and the results were expressed as the percentages of each fatty acid in the total fatty acids. The same sample was analyzed in triplicate under the same conditions.

### 2.4. Flesh Color and Texture Determination

The color of the fish skin and muscle was represented by L*, a*, and b* values and was assessed using a colorimeter (Konica Minolta, Shanghai, China); tests were performed in triplicate. After the fish were anesthetized, the moisture was removed from their surfaces. The middle area of the body was selected for color measurements, which were performed in triplicate. Flesh in the same position was subjected to pH measurements in triplicate using a pH tester (pH-STAR, Matthaus, Germany). To investigate flesh texture, a portion of the dorsal muscle (1 cm^3^) around the lateral line in the middle of the body was collected and tested using a CT3 Texture Analyzer (Brookfield Engineering Laboratories, Inc., Brookfield, WI, USA). To measure cooking loss, the sample (approximately 3 g) was placed into a retort pouch in an 80 °C water bath; the surface moisture was then wiped with filter paper after 6 min. The cooking loss was calculated as follows: Cooking loss = (W1 − W2)/W1 × 100%, where W1 and W2 (g) are the weight of sample before cooking and after cooking.

### 2.5. Volatile Compound Analysis

The volatile organic compounds in the samples were determined using a FlavourSpec^®^Flavour headspace-gas-chromatogramphyion-mobility (HS-GC-IMS) spectrometry instrument (Haineng Scientific Instrument, Jinan, China). Based on the steps described in the work of Man et al. [25], 1.5 g of sample (chopped into minced meat) and 0.1 μg (2.0 μL) of 2-methyl-3-heptanone were placed into a headspace vial and incubated for 15 min at 60 °C; the sample was then automatically injected by the device with the syringe, and injector temperatures were maintained at 85 °C and 45 °C, respectively. The GC was separated at 60 °C through an MXT-5 capillary column (15 m × 0.53 mm; 1 μm), and the temperature of the GC column was also set at 60 °C with nitrogen. Meanwhile, research-grade nitrogen was employed as the carrier gas for 0–2 min at 2 mL/min, followed by 2–20 min at 100 mL/min. Additionally, the temperature of the drift was set at 45 °C with a 9.8 cm long drift tube, and the drift gas was selected as nitrogen (150 mL/min). C4-C9 n-ketones obtained from Sinopharm Chemical Reagent Beijing Co., Ltd. (Beijing, China) were used as references to determine retention indexes. The same sample was measured in triplicate under the same conditions.

### 2.6. Collagen Content Analysis

The flesh collagen content (type I) was assessed using a commercial ELISA kit (Nanjing Jiancheng Bioengineering Institute, Nanjing, China). Before the test, approximately 100 mg of the sample was homogenized in an ice bath in pre-cooled phosphate-buffered solution (*w*/*v*, 1:9; Beijing Solarbio Science & Technology Co., Ltd., Beijing, China). The mixture was then centrifuged at 5000× *g* for 10 min at 4 °C, and the supernatant was used for collagen content detection according to the manufacturer’s instruction. The corresponding antibody was added to the collagen sample; then, the recognition antigen labeled with horseradish peroxidase was added. After incubation for 30 min at 37 °C without light, the horseradish peroxidase was added to catalyze tetramethyl-benzidine into a blue color. The blue color turned yellow, and the absorbance was measured at 450 nm (Thermo Fisher Scientific Co., Ltd., Shanghai, China).

### 2.7. Hematoxylin and Eosin (H&E) Staining

The details on HE staining are described elsewhere, in the work of Zhang et al. [26]. First, the fixed samples were dehydrated in 75, 80, 90, 95, and 100% anhydrous ethanol. The samples were then subjected to a 1:1 (*v*/*v*) mixture of 100% anhydrous ethanol and xylene and were embedded in paraffin using an embedding machine (Junjie Electronics Co., Ltd., Wuhan, China). Next, 4 μm sections were obtained using a paraffin slicer (Leica Instrument Co., Ltd., Shanghai, China). Before staining, the slices were dewaxed using a dewaxing solution, anhydrous ethanol, and 75% alcohol, in turn. Specific staining was performed based on the recommended instructions (Beyotime Biotechnology Co., Ltd., Beijing, China). Histological observations were made using a ZEISS microscope (Axio Scope, A1, Oberkochen, Germany). Moreover, to observe muscle fiber diameter, 60 muscle fibers per group were selected and measured using the Image-J software (v1.54, National Institutes of Health, Bethesda, MD, USA).

### 2.8. Free Amino Acids Profile Analysis

The flesh free amino acid composition was determined as described by Tu et al. [11]. The sample (approximately 300 mg) was homogenized in 0.01 mol/L hydrochloric acid and then incubated at 4 °C for 1 h. After centrifugation (2500× *g*) at 4 °C for 5 min, the supernatant was collected and filtered through a 0.22 μm membrane. Subsequently, the obtained solution was mixed with an equal volume of sulfosalicylic acid (8%) before storage at 4 °C overnight. The supernatant used to perform the free amino acid profile analysis in triplicate was obtained by centrifugation and filtering, as described above.

### 2.9. Flavor Nucleotide Detection

An analysis of flesh flavor nucleotide content was performed as described by Tao et al. [27]. Approximately 500 mg of the sample was homogenized in trichloroacetic acid (5% by vol) and then incubated on ice for 2 h. Then, the mixture was filtered through a 0.22 μm hydrophilic polyether sulfone filter membrane and centrifugated (4 °C) at 12,000× *g* for 10 min. The supernatants were collected based on these procedures. The remaining pellets were re-extracted using trichloroacetic acid and the supernatant was also obtained, as described above. The pH of the supernatants was adjusted to 5.75, and the volume was set to 50 mL using a volumetric bottle. The filtered liquid (0.22 μm hydrophilic polyether sulfone filter membrane) was collected and used for testing via high-performance liquid chromatography (HPLC, Agilent Technologies, Santa Clara, CA, USA); test were performed in triplicate.

### 2.10. Electronic Tongue Evaluation

The fresh sample was first homogenized in pre-cooled ultra-pure water, before being centrifugated (4 °C) at 12,000× *g* for 10 min. The supernatant was used to perform an electronic tongue evaluation (in triplicate). The detection system (α-ASTREE, Alpha MOS, France) was equipped with an array of six cross-sensitive potential sensors that responded to the six basic tastes, including sour, sweet, bitter, salty, umami, and mellow.

### 2.11. RNA Isolation and Gene Expression Analysis

Total RNA isolation and cDNA synthesis were performed using commercial kits (Takara Biomedical Technology Co., Ltd., Dalian, China); RNA samples were quantified using a nucleic acid quantifier (Thermo Fisher Scientific Co., Ltd., Shanghai, China), and quality was controlled by agarose gel electrophoresis. Gene amplification was performed in triplicate using a quantitative real-time polymerase chain reaction system (Applied Biosystems, Foster City, CA, USA). The reaction solution consisted of 0.5 μL cDNA (25 ng), 0.4 μL forward primer, 0.4 μL reverse primer, 8.7 μL sterilized ultrapure water, and 10 μL 2 × SYBR Green Premix (Xinkailai Biotechnology Co., Ltd., Guangzhou, China). The reaction procedure commences with an initial denaturation at 95 °C for 30 s, followed by a denaturation phase at 95 °C for 10 s, annealing at 60 °C for 10 s, and extension at 72 °C for 30 s for 40 cycles. Finally, a melting curve was constructed to confirm that only the signal was amplified. The comparative CT method (2^−ΔΔCt^) [28,29] was used to perform the data analysis, and the corresponding primers are provided in Supplementary Table S2.

### 2.12. Enzyme Activity Analysis

Muscular alanine aminotransferase (ALT) (Nanjing jiancheng Bioengineering Institute, Nanjing, China) and δ^1^-pyrrolin-5-carboxylate synthase (PC5S) (Beijing Solarbio Science & Technology Co., Ltd., Beijing, China) activities were measured using commercial kits. The ALT catalyzed the production of pyruvate and glutamic acid (Glu) at 37 °C and pH 7.4. Then, 2, 4-dinitrophenylhydrazine was added after 30 min to produce pyruvate phenylhydrazone (a red-brown color). Specifically, the sample (approximately 50 mg) was homogenized in an ice bath in normal saline solution (*w*/*v*, 1:9; Beijing Solarbio Science & Technology Co., Ltd., Beijing, China). The mixture was then centrifuged at 2500× *g* for 10 min at 4 °C, and the supernatant was used for enzyme activity detection according to the manufacturer’s instruction. Finally, the absorbance was measured at 505 nm (Thermo Fisher Scientific Co., Ltd., Shanghai, China). The PC5S catalyzed the phosphorylation of Glu and the reduction of Glu γ-semialdehyde in the presence of NADPH (0.2 mM) and ATP (3 mM) at 37 °C and pH 7.4. The PC5S activity was calculated based on the absorbance of NADPH at 340 nm (Thermo Fisher Scientific Co., Ltd., Shanghai, China). Briefly, approximately 50 mg of the sample was homogenized in an ice bath in extraction solution I (*w*/*v*, 1:5); then, extraction solution II (10 μL) was added and centrifuged at 8000× *g* for 15 min at 4 °C. The extraction solutions were provided with the kit. The supernatant was used for enzyme activity based on the recommended steps.

### 2.13. Data Calculation and Statistical Analysis

#### 2.13.1. Data Calculation

The equivalent umami concentration (EUC) value was calculated as follows: ΣAiBi + 1218(Σ AiBi) (Σ AjBj) [20]. Ai and Aj represent the contents of the umami amino acid and flavor nucleotides, respectively. Bi and Bj denote the relative umami coefficients of the umami amino acid and flavor nucleotides, respectively. The taste active value (TAV) of the free amino acid was calculated as the concentration of the amino acid/taste threshold value [11]. Analogously, the odor-active value (OAV) of the volatile substances was calculated as the concentration of the volatile substance/olfactory threshold value [11]. The ΔE value was calculated as follows: $\sqrt{\left(\Delta L*\Delta L+\Delta a*\Delta a+\Delta b*\Delta b\right)}$, in which ΔL, Δa, and Δb are the differences in L, a, and b values between the two groups, respectively [30].

#### 2.13.2. Statistical Analysis

An independent sample t-test (PASW Statistics 18, Chicago, IL, USA) was used to analyze the results. The data were expressed as mean ± standard deviation, and statistical significance was set at *p* ≤ 0.05. Beforehand, normality and homoscedasticity tests were performed. In addition, PCA was conducted in PASW Statistics 18 software (Chicago, IL, USA). All items were standardized prior to analysis. The Kaiser–Meyer–Olkin test and Bartlett’s test of sphericity were performed to confirm the suitability for analysis. Components with eigenvalues >1 were retained.

## 3. Results

### 3.1. Nutritional Quality

#### 3.1.1. Proximate Composition

Crude protein, crude fat, ash, and moisture contents are displayed in Table 1. No prominent differences were observed in the crude protein, moisture, or ash contents between the two populations. However, the crude fat content of the salt-tolerant population was lower than that of the control population.

#### 3.1.2. Fatty Acid Profile

**Figure 1:**
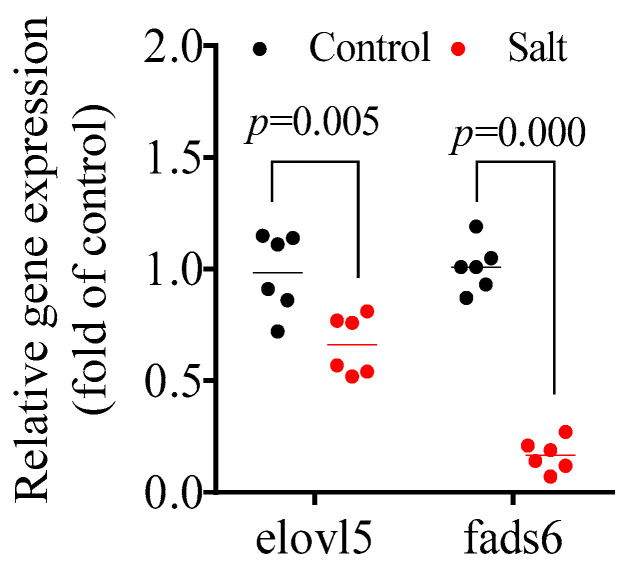
Muscular gene expression involved in LC-PUFA synthesis. *p* ≤ 0.05 indicates a significant difference.

The fatty acid composition is shown in Table 2. Specifically, the contents of C16:0, C18:1n-7, C20:3n-6, C20:5n-3, and C22:6n-3 decreased in the salt-tolerant population compared to the control population. Nevertheless, the inverse results were observed for the C14:0, C16:1n-7, C18:1n-9, C18:2n-6, and C18:3n-3 contents. No obvious differences were observed for C18:0, C18:3n-6 (not detected), C20:4n-6, and C22:4n-6. Based on the fatty acid classification, the salt-tolerant population had a higher monounsaturated fatty acid (MUFA) content, but lower contents of saturated fatty acid (SFA), polyunsaturated fatty acid/long-chain polyunsaturated fatty acid (PUFA/LC-PUFA), docosahexaenoic acid (DHA), and eicosapentaenoic acid (EPA). Muscular genes involved in EPA and DHA synthesis, including fatty acid elongase 5 (elovl5) and fatty acid desaturase 6 (fads6), were also measured. The data showed that these two genes were notably downregulated in the salt-tolerant population compared to the control population (Figure 1).

### 3.2. Sensory Value Parameters

#### 3.2.1. Color Parameters

The b* value of the dorsal skin in the salt-tolerant population was lower than that in the control population, and the a* and L* values were not affected (Table 3). In the dorsal muscles and abdominal skin, the L* value was higher in the salt-tolerant population than the control population, but the opposite results were observed for the changes in the a* and b* values. In addition, the pH values of the dorsal muscles of the salt-tolerant population were notably lower than those of the control population. The ΔE values of the dorsal muscle and abdominal skin were greater than 6.

#### 3.2.2. Odor Substance

**Figure 2:**
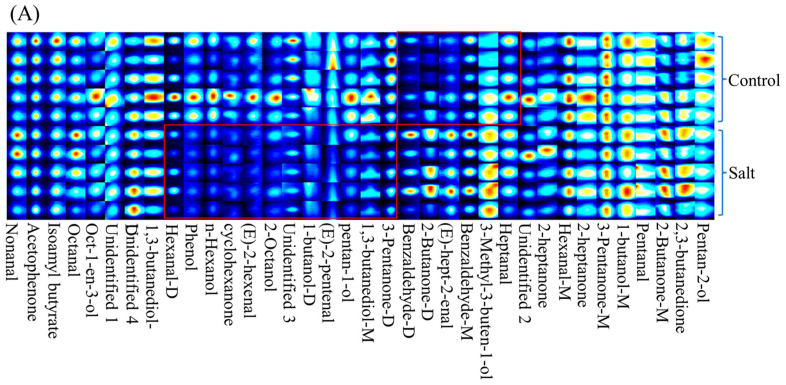

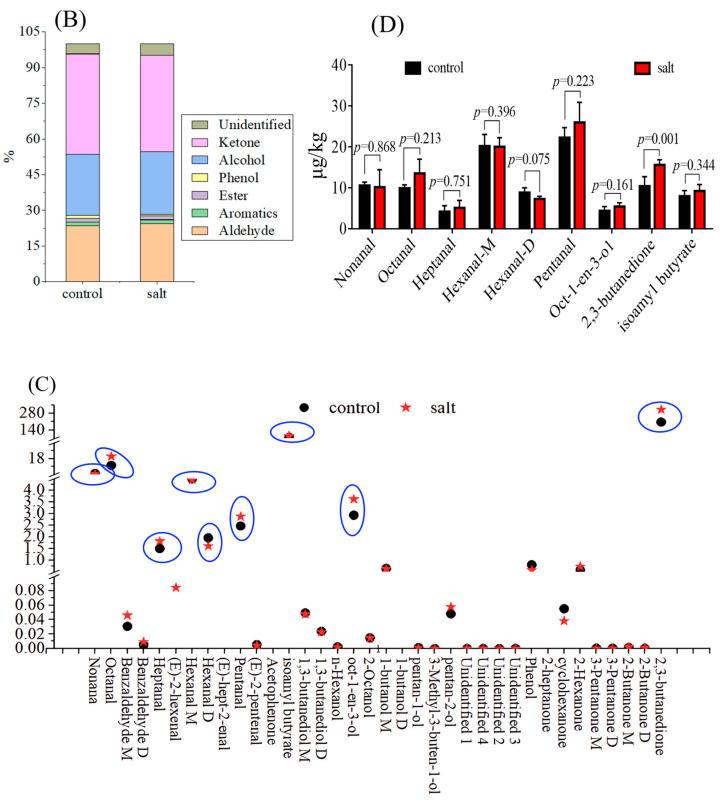
Volatile substance contents of fish (n = 6). *p* ≤ 0.05 indicates a significant difference. (**A**): volatile substance fingerprint; (**B**): volatile substances categories; (**C**): OAVs of volatile compounds; (**D**): volatile substance content with OAV ≥ 1.

A total of 36 volatile compounds, grouped into seven categories, were detected (Table 4). In general, volatile compounds were distributed differently between the two populations based on their fingerprint (Figure 2A). Of these categories, ketones were the most abundant, followed by alcohols and aldehydes, and the 3-Pentanone (monomer and dimer) content was the highest among these compounds in both populations (Figure 2B; Table 4). There were nine volatile compounds with OAV ≥1 in both populations, including nonanal, octanal, heptanal, hexanal-M, hexanal-D, Oct-1-en-3-ol, pentanal, 2,3-butanedione, and isoamyl butyrate (Figure 2C). However, only the 2,3-butanedione content differed between the two populations, and it was higher in the salt-tolerant population (Figure 2D).

### 3.3. Flesh Texture

**Figure 3:**
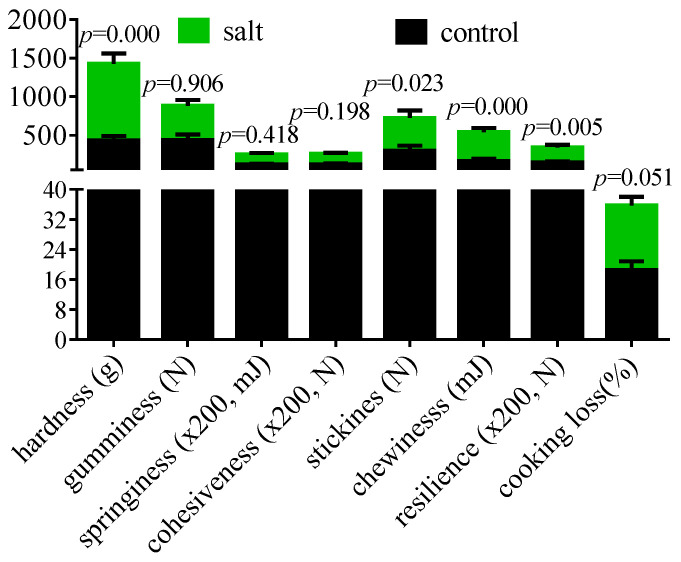
Flesh texture of fish (n = 6). *p* ≤ 0.05 indicates a significant difference.

**Figure 4:**
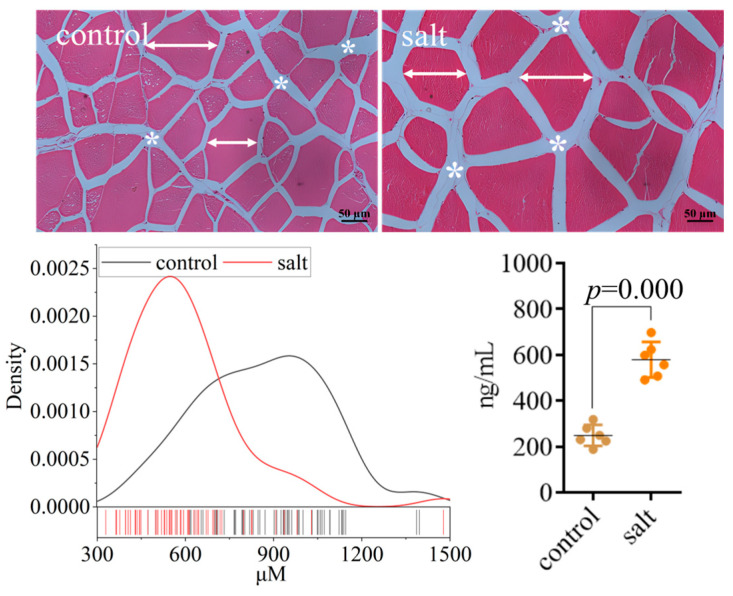
Morphological analysis and collagen I content of fish (n = 6). *p* ≤ 0.05 indicates a significant difference (straight line with arrows: cell diameter; asterisk: cellular space).

The flesh texture parameters are displayed in Figure 3. The muscular hardness, stickiness, chewiness, resilience, and cooking loss increased in the salt-tolerant population. However, the gumminess, springiness, and cohesiveness remained stable. The collagen content of the flesh is exhibited in Figure 4. These data indicated that flesh collagen content was markedly increased in the salt-tolerant population. Moreover, the morphology showed an increased space between myocytes and a decreased cell diameter in this population (Figure 4).

### 3.4. Flesh Flavor Index

#### 3.4.1. Free Amino Acids

**Figure 5:**
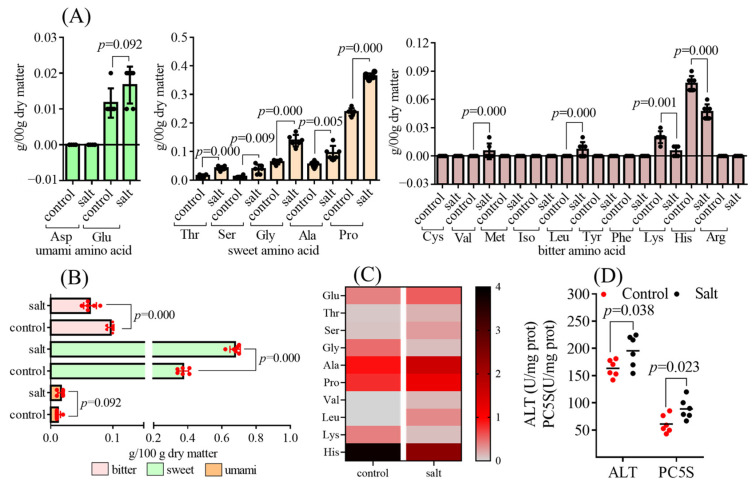
Free amino acid contents of fish (n = 6). *p* ≤ 0.05 indicates a significant difference). (**A**): free amino acid contents; (**B**): free amino acid classification; (**C**): heat map distribution of free amino acids with TAV > 1; (**D**): enzyme activity.

A total of seventeen free amino acids were analyzed, of which aspartic acid (Asp), cysteine (Cys), methionine (Met), isoleucine (Iso), tyrosine (Tyr), phenylalanine (Phe), and arginine (Arg) were not detected because of their low content outside the detection threshold (Figure 5A). Based on the classification, bitter amino acid content was obviously decreased in the salt-tolerant population, and the opposite result was observed for sweet amino acid contents. The two populations had similar umami amino acid contents (Figure 5B). In addition, ten free amino acids with a TAV > 1 were noted, including Glu, threonine (Thr), serine (Ser), glycine (Gly), alanine (Ala), proline (Pro), valine (Val), leucine (Leu), lysine (Lys), and histidine (His) (Figure 5C). The TAV of the free amino acid is listed in Supplementary Table S3. The salt-tolerant population had higher Thr, Ser, Gly, Ala, Pro, Val, and Leu contents than the control population, and the opposite results were observed for Lys and His contents. No obvious differences were found in Glu contents between the two populations (Figure 5A). Collectively, Ala, Pro, and His were the main amino acids responsible for flavor presentation based on the TAV results (Figure 5C). Except for His, which is an essential amino acid in fish, key enzyme activities that influenced Ala and Pro synthesis were detected. As excepted, the muscular ALT and PC5S activities were enhanced in the salt-tolerant population compared to those in the control group (Figure 5D).

#### 3.4.2. Flavor Nucleotides

**Figure 6:**
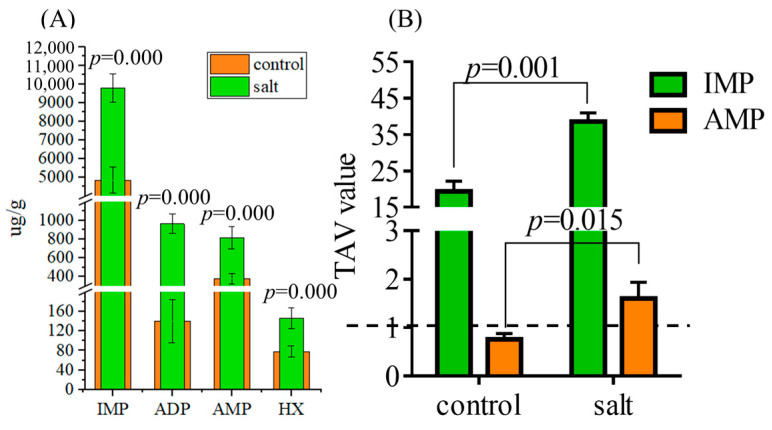
Flavor nucleotide contents of fish (n = 6, μg/g dry matter). *p* ≤ 0.05 indicates a significant difference. (**A**) Flavor nucleotide contents; (**B**) TAVs of flavor nucleotides. Data in parentheses indicate the threshold.

Figure 6 shows the flavor nucleotide contents in both populations, including inositol phosphate (IMP), adenosine diphosphate (ADP), adenosine monophosphate (AMP), and hypoxanthine (HX). The contents of the four flavor nucleotides were higher in the salt-tolerant population than in the control group (Figure 6A). In addition, IMP and AMP demonstrated TAVs >1, and higher TAVs were observed in the salt-tolerant population (Figure 6B).

#### 3.4.3. Equivalent Umami Concentration and Electronic Tongue Analysis

**Figure 7:**
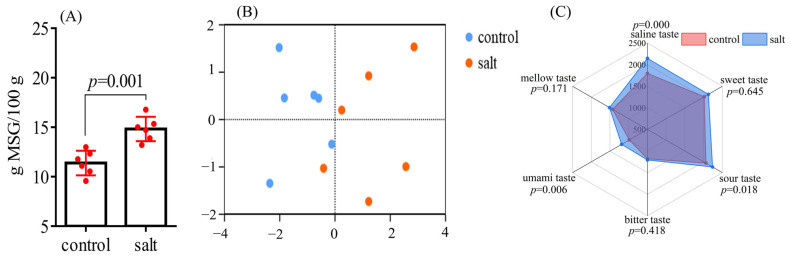
Equivalent umami concentration and electronic tongue analysis of fish (n = 6). *p* ≤ 0.05 indicates a significant difference. (**A**): EUC of samples; (**B**): PCA analysis; (**C**): radar map of flavor substances.

The results in Figure 7A reveal that the EUC values were different between the two populations. Higher EUC values were detected in the salt-tolerant population. The electronic tongue was equipped with six sensors to sense the saline, sweet, sour, bitter, umami, and mellow tastes of the samples. PCA analysis showed a significant difference in flesh flavor between the two populations (Figure 7B). Specifically, flesh samples from the salt-tolerant population had higher salinities, as well as sour and umami tastes, compared to those from the control population (Figure 7C).

## 4. Discussion

Body composition is a basic index that reflects the nutritional quality of the flesh [32]. Here, the crude protein, ash, and moisture contents showed no significant differences between the two populations, which is consistent with a previous report on *S. paramamosain* [20]. To maintain the balance of osmotic pressure, more energy is expended on the uptake and secretion of ions [33], which may have contributed to the decline in the lipid and SFA content of the salt-tolerant population. Lipids are the primary energy source preferred by fish [34], and SFA are more prone to β-oxidation than MUFA and PUFA [35]. In addition, increased MUFA content, decreased LC-PUFA contents, and the expected downregulation of elovl5 and fads6 gene expressions in the salt-tolerant population were observed, which are consistent with the results of *Oreochromis niloticus* [9] and *Paralichthys orbignyanus* [36]. Notably, there were increases in DHA and EPA contents after culturing in salt water [37], which may have resulted from species differences or the time of saltwater culturing.

The sensory value of fish, including meat color and odor, is an important factor that affects the purchasing desires of consumers. Regarding the color index, both dorsal muscle and abdominal skin had an ΔE > 6 in the present study, indicating a significant color separation between the two populations [38]. During prolonged storage, the deterioration of fish flesh quality is mainly caused by fat oxidation and protein denaturation, resulting in decreased hardness and chewiness, as well as darkening and a gradual shift to a yellow-green color [39]. Based on this result, higher L* values (brighter) and lower b* values (+b*: yellow; −b*: blue) were beneficial to flesh sensory quality [30]. The change in energy consumption under saltwater led to a change in myoglobin content, which contributed 80–90% to muscle color [40] and led to a decreased a* value (+a*: red, −a*: green) of the abdominal skin and dorsal muscle in the salt-tolerant population.

Regarding odor characteristics, ketones, alcohols, and aldehydes were the most abundant, indicating that they represent the main volatile substances affecting meat smell. These characteristics have also been identified in other aquatic animals [41]. The 3-pentanone content was the highest among the volatile substances, giving the meat a fruity flavor [42]. The substances with an OAV ≥1 are recognized as the main contributors to the overall aroma [43]. Among others, the majority were straight-chain aldehydes with a fishy odor (e.g., nonanal and hexanal), and several compounds (e.g., oct-1-en-3-ol and esters) had a refreshingly sweet flavor [44,45]. This result is consistent with the perception that most aquatic products have a fishy and earthy taste. Significantly, 2,3-butanedione, with the highest OAV, was higher in the salt-tolerant population, thereby imparting a desirable creamy aroma to the salt-tolerant population [46].

Flesh texture is critical to meat quality and is positively affected by the collagen content [47]. In this study, a higher collagen content was observed in the salt-tolerant population, which is inconsistent with the results of short-term salinity stress in *M. salmoides* [48], indicating that the duration of saline acclimation induced differential effects on collagen, and the underlying mechanisms still require further investigation. The myocyte diameter was correlated with hardness, resilience, chewiness, and even meat juice loss [49,50]. Fish meat with higher hardness, chewiness, and chewiness is considered to have a better taste [51], indicating that the salt-tolerant population in this study had a better taste. Moreover, the increased cooking loss in the salt-tolerant population aligns with the earlier report of Li et al. [52].

The free amino acid composition of meat directly affects its flavor [53], and amino acids with TAV > 1 were recognized as the primary flavor contributor [54]. Overall, the salt-tolerant population had a lower bitter taste and higher sweet-tasting free amino acid content, which helped improve flesh quality. Among the primary contributors to flavor, Ala (sweet), Pro (sweet), and His (bitter) were the top three compounds. Although the salt-tolerant population had higher levels of Ala and Pro and lower concentrations of His, the sweet taste measured by the electronic tongue was stable between the two populations. The 5′-nucleotides affected flesh taste through the synergistic effect with free amino acids [55], and the elevated Hx (bitter) content may have integrated the sweetness in the salt-tolerant population. As expected, the enzymatic activities of ALT and PC5S, which influenced the synthesis of Ala and Pro, respectively, were higher in the salt-tolerant population. On the other hand, the four detected nucleotides, including IMP, ADP, AMP, and Hx, were higher in the salt-tolerant population than those in the control population. The TAV of IMP was greater than 1 in both populations, implying that it was the primary contributor to the umami taste in *M. salmoides*. The predominant taste in nucleotides varied among the species. IMP and AMP reportedly constitute the primary flavor nucleotides in white shrimp [56], whereas only IMP has been found in sturgeon [57]. In addition to IMP, the TAV of the salt-tolerant population AMP was greater than 1 and higher than that of the control population, which also contributed to the distinct flesh quality of the salt-tolerant population. Collectively, the umami intensity of the new population was improved based on the EUC data, which is a comprehensive parameter reflecting the effects of free amino acids and 5′-nucleotides on flesh flavor [11].

An electronic tongue analysis is an effective reflection of meat flavor. In the PCA, these samples were clearly separated, indicating a distinct difference in flavor between the two populations. The higher saline taste of the salt-tolerant population may have resulted from the change in the ion concentration required for the maintenance of osmotic pressure in salt water [33]. The increased sour taste was consistent with the decreased pH in the salt-tolerant population. To adapt to a high-salinity water environment, glycolysis was enhanced to meet increased energy requirements [58,59], and the resulting lactic acid may lead to an increased sour taste. Flavor nucleotides and free amino acids have synergistic freshening effects (e.g., IMP and glu) [60]. Considering the higher levels of IMP, AMP, and Glu in the salt-tolerant population, an increased umami taste was expected. The unaffected sweet, bitter, and mellow tastes may represent a combined result of different flavor substance contents. For instance, both sweet amino acid and bitter nucleotide contents were elevated in the salt-tolerant population.

## 5. Conclusions

Long-term saline adaptation affects flesh quality in a novel salt-tolerant *M. salmoides* population. Nutritional analysis revealed stable crude protein, moisture, and ash content, but reduced fat, SFA, and LC-PUFA, alongside increased MUFA in the new population. Though dorsal skin coloration remained unchanged, the meat color of the abdominal skin and dorsal muscle of the selected salt-tolerant population was brighter and fresher, with reduced redness. In addition, this new population developed a distinctive creamy aroma due to the elevated 2,3-butanedione levels, complementing the fishy and earthy taste derived from straight-chain aldehydes. The new population exhibited different textural properties, including increased hardness, stickiness, chewiness, and resilience, alongside higher collagen I content and wider myocyte spacing. The combined effects of altered flavor compounds (e.g., increased sweet amino acids and bitter nucleotides) resulted in the meat of the selected salt-tolerant population tasting saltier, fresher, and sourer. Of course, follow-up studies should incorporate direct acceptability assessments of consumers to provide reference for the potential development of this new population as an alternative aquaculture strain.

## Figures and Tables

**Table 1:** Proximate composition of fish (n = 6). Different letters indicate significant differences (*p* ≤ 0.05, g/100 g).

	Control	Salt		Control	Salt
Crude protein	19.55 ± 0.84	19.80 ± 0.98	moisture	77.76 ± 1.10	78.82 ± 0.90
Crude fat	2.84 ± 0.64 ^a^	1.96 ± 0.50 ^b^	ash	1.25 ± 0.09	1.24 ± 0.10

**Table 2:** Fatty acid composition of fish (n = 6, % of total fatty acids) ^1^.

	Control	Salt		Control	Salt
C14:0	1.10 ± 0.18 ^b^	1.39 ± 0.11 ^a^	C18:3n-3	1.19 ± 0.26 ^b^	1.55 ± 0.14 ^a^
C16:0	24.39 ± 1.83 ^a^	20.97 ± 1.03 ^b^	C20:1n-9	0.36 ± 0.11	0.47 ± 0.13
C16:1n-7	3.05 ± 0.72 ^b^	4.40 ± 0.70 ^a^	C20:3n-6	3.91 ± 0.58 ^a^	2.02 ± 0.24 ^b^
C18:1n-7	2.76 ± 0.61 ^a^	1.92 ± 0.27 ^b^	C20:4n-6	0.88 ± 0.23	0.95 ± 0.23
C18:0	0.23 ± 0.08	0.27 ± 0.07	C20:5n-3	6.14 ± 1.67 ^a^	1.86 ± 0.25 ^b^
C18:1n-9	18.81 ± 1.50 ^b^	32.73 ± 1.38 ^a^	C22:4n-6	0.27 ± 0.11	0.29 ± 0.08
C18:2n-6	10.67 ± 0.94 ^b^	24.57 ± 0.97 ^a^	C22:5n-3	2.70 ± 0.51 ^a^	0.95 ± 0.24 ^b^
C18:3n-6	0.00 ± 0.00	0.00 ± 0.00	C22:6n-3	22.67 ± 1.43 ^a^	8.29 ± 0.80 ^b^
	control		salt		
SFA	25.72 ± 1.85 ^a^		22.63 ± 1.00 ^b^		
MUFA	25.00 ± 1.92 ^b^		39.52 ± 1.83 ^a^		
PUFA/LC-PUFA	48.41 ± 1.08 ^a^		40.48 ± 1.32 ^b^		
DHA + EPA	28.81 ± 1.15 ^a^		10.15 ± 0.93 ^b^		

^1^ Different letters indicate significant differences (*p* ≤ 0.01). SFA: C14:0, C16:0, C18:0; MUFA: C16:1n-7, C18:1n-7, C18:1n-9, C20:1n-9; PUFA/LC-PUFA: C18:2n-6, C18:3n-6, C18:3n-3, C20:3n-6, C20:4n-6, C22:4n-6, C20:5n-3 (DHA), C22:5n-3, C22:6n-3 (EPA).

**Table 3:** Color and pH indexes of fish (n = 6). *p* ≤ 0.05 indicates a significant difference.

Tissue		Control	Salt	*p*-Value
Dorsal skin	L*	30.46 ± 0.89	30.21 ± 1.83	0.773
	a*	−2.33 ± 0.83	−2.56 ± 0.39	0.543
	b*	7.14 ± 1.03	4.29 ± 0.54	0.000
ΔE	4.25 ± 0.88			
Dorsal muscle	L*	41.93 ± 1.10	45.53 ± 1.96	0.003
	a*	2.55 ± 0.45	−0.71 ± 0.43	0.000
	b*	0.68 ± 0.46	−1.51 ± 0.42	0.000
ΔE	8.23 ± 1.8			
Abdominal skin	L*	79.41 ± 1.38	82.47 ± 2.05	0.024
	a*	2.55 ± 1.01	0.85 ± 0.39	0.006
	b*	10.89 ± 1.92	6.51 ± 1.26	0.003
ΔE	6.42 ± 0.82			
Dorsal muscle	pH	7.48 ± 0.28	7.04 ± 0.21	0.011

**Table 4:** Volatile compounds identified in *M. salmoides* flesh (m: Monomer; D: dimer; μg/kg).

Category	Compound	Control	Salt	Formula	Odor Threshold
Aldehyde	Nonanal	10.59 ± 0.81	10.19 ± 4.27	C_9_H_18_O	1.1
	Octanal	9.92 ± 0.84	13.51 ± 3.50	C_8_H_16_O	0.7
	Benzaldehyde-M	10.80 ± 1.59	16.15 ± 3.03	C_7_H_6_O	350
	Benzaldehyde-D	1.87 ± 0.46	3.25 ± 0.42	C_7_H_6_O	350
	Heptanal	4.23 ± 1.42	5.11 ± 1.83	C_7_H_14_O	2.8
	(E)-2-hexenal	2.13 ± 0.41	1.63 ± 0.36	C_6_H_10_O	19.2
	Hexanal-M	20.23 ± 2.81	20.04 ± 2.2	C_6_H_12_O	4.5
	Hexanal-D	8.85 ± 1.17	7.29 ± 0.67	C_6_H_12_O	4.5
	(E)-2-pentenal	8.20 ± 1.35	6.44 ± 1.09	C_5_H_8_O	1500
	Pentanal	22.28 ± 2.45	25.97 ± 4.90	C_5_H_10_O	9
	(E)-hept-2-enal	3.43 ± 0.73	5.70 ± 0.89	C_7_H_12_O	13
Alcohol	Oct-1-en-3-ol	4.41 ± 1.03	5.44 ± 0.95	C_8_H_16_O	1.5
	2-Octanol	4.28 ± 1.03	4.21 ± 0.57	C_8_H_18_O	290
	n-Hexanol	6.05 ± 1.87	4.68 ± 1.13	C_6_H_14_O	2500
	1,3-butanediol-M	11.37 ± 0.93	11.00 ± 1.47	C_4_H_10_O_2_	230
	1,3-butanediol-D	5.50 ± 0.78	5.31 ± 0.57	C_4_H_10_O_2_	230
	Pentan-1-ol	5.21 ± 1.45	4.28 ± 1.12	C_5_H_12_O	4000
	1-butanol-M	24.98 ± 1.73	23.98 ± 4.24	C_4_H_10_O	38
	1-butanol-D	5.79 ± 0.56	4.27 ± 0.46	C_4_H_10_O	38
	3-Methyl-3-buten-1-ol	22.29 ± 2.69	36.62 ± 4.85	C_5_H_10_O	/
	Pentan-2-ol	13.98 ± 1.39	16.74 ± 1.32	C_5_H_12_O	290
Ketone	2-heptanone	2.21 ± 0.86	2.43 ± 0.64	C_7_H_14_O	6.8
	Cyclohexanone	5.53 ± 0.98	3.84 ± 0.84	C_6_H_10_O	100
	2-Hexanone	3.95 ± 0.35	5.06 ± 0.83	C_6_H_12_O	6.8
	3-Pentanone-M	60.80 ± 7.10	72.80 ± 7.91	C_5_H_10_O	200,000
	3-Pentanone-D	75.76 ± 11.44	56.18 ± 9.76	C_5_H_10_O	200,000
	2-Butanone-M	21.13 ± 2.76	33.71 ± 3.87	C_4_H_8_O	16,000
	2-Butanone-D	3.44 ± 1.04	14.30 ± 1.36	C_4_H_8_O	16,000
	2,3-butanedione	10.42 ± 2.33	15.6 ± 1.30	C_4_H_6_O_2_	0.05
Ester	Isoamyl butyrate	7.97 ± 1.37	9.26 ± 1.57	C_9_H_18_O_2_	0.1
Phenol	Phenol	4.55 ± 1.03	3.29 ± 0.85	C_6_H_6_O	5.6
Aromatics	Acetophenone	7.48 ± 0.79	6.50 ± 1.42	C_8_H_8_O	65
Unidentified	Unidentified 1	4.14 ± 1.12	4.05 ± 0.65	-	/
	Unidentified 2	4.73 ± 0.31	4.42 ± 0.87	-	/
	Unidentified 3	3.93 ± 0.53	3.91 ± 0.68	-	/
	Unidentified 4	9.80 ± 1.18	12.09 ± 0.90	-	/

The odor threshold value was referred to in a related study [31] and an online website (http://www.chemicalbook.com/ProductIndex.aspx (accessed on 9 April 2024)).

## Data Availability

The original contributions presented in this study are included in the article/Supplementary Materials. Further inquiries can be directed to the corresponding author.

## Supplementary Materials

The following supporting information can be downloaded at https://www.mdpi.com/article/10.3390/foods14183180/s1. Supplementary Table S1: Weights of fish and samples used for parameters analysis. Supplementary Table S2: Primers used for qRT-PCR. Supplementary Table S3: Taste threshold values of free amino acids.
