# Supplementary material for: Long-Term Saline Water Adaptation Alters the Meat Quality of Micropterus salmoides from a New Salt-Tolerant Population
Source: Foods. 2025 Sep 12;14(18):3180. doi: 10.3390/foods14183180 (PMC12470199; doi:10.3390/foods14183180)
Supplement: Supplementary file 1 [file foods-14-03180-s001.zip › foods-3708429-supplementary.pdf]

Table S1. Weights of fish and samples used for parameters analysis

	Salt-tolerant population	Control population		Salt-tolerant population	Control population		Salt-tolerant population	Control population
fish weight	601 g	599 g	sample weight used for cooking loss determination	2.34 g	2.71 g	sample weight used for electronic tongue analysis	5.51 g	4.56 g
	611 g	609 g		2.77 g	2.44 g		5.34 g	5.03 g
	605 g	603 g		3.21 g	3.22 g		5.98 g	4.88 g
	603 g	610 g		2.94 g	2.95 g		4.79 g	5.54 g
	607 g	605 g		3.05 g	3.13 g		4.94 g	5.70 g
	605 g	605 g		3.51 g	3.07 g		5.11 g	4.21 g

Table S2. Primers used for qRT-PCR

gene	Forward (5’-3’)	Reverse (5’-3’)	Accession Number	Product length
Elovl5	GCCGTACCTTTGGTGGAAGA	TGAAAGCACAGCCATCCCAT	XM_038737607.1	126 bp
Fads6	CCAAGGAAGGACAGACGCTT	CTGTGAGGTGACAGGAACCC	XM_038735778.1	169 bp
β-actin	TCCGGTATGTGCAAAGCTGG	TCTGGGACGTCCAACAATGG	MH018565.1	78 bp

Table S3. Taste threshold values of free amino acids

Free amino acids	Threshold (mg/100g)
Aspartic acid	50
Glutamic acid	30
Threonine	/
Serine	150
Glycine	130
Alanine	60
Proline	300
Cystine	/
Valine	40
Methionine	/
Isoleucine	90
Leucine	190
Tyrosine	/
Phenylalanine	90
Lysine	50
Histidine	20
Arginine	50
